# How an increase in income affects the use of dental care services among a low-income population: evidence from the Finnish basic income experiment

**DOI:** 10.1186/s12913-024-10933-0

**Published:** 2024-04-22

**Authors:** Miska Simanainen

**Affiliations:** 1 Department of Sociology, Stockholm University, Universitetsvägen 10B, Floor 8 and 9, S-106 91, Stockholm, Sweden; 2 Social Insurance Institution of Finland, Helsinki, Finland

**Keywords:** Cash transfers, Income, Dental care, Policy experiment, Service usage

## Abstract

**Background:**

Previous research has shown that the use of dental care services has a significant socioeconomic gradient. Lower income groups tend to use dental care services less, and they often have poorer dental health than higher income groups. The purpose of this study is to evaluate how an increase in income affects the use of dental care services among a low-income population.

**Methods:**

The study examines the causal effect of increasing cash transfers on the use of dental care services by utilizing unique register-based data from a randomized field experiment conducted in Finland in 2017–2018. The Finnish basic income experiment introduced an exogenous increase in the income of persons who previously received basic unemployment benefits. Register-based data on the study population’s use of public and private dental care services were collected both for the treatment group (*N* = 2,000) and the control group (*N* = 173,222) of the experiment over a five-year period 2015–2019: two years before, two years during, and one year after the experiment. The experiment’s average treatment effect on the use of dental care services was estimated with OLS regressions.

**Results:**

The Finnish basic income experiment had no detectable effect on the overall use of dental care services. However, it decreased the probability of visiting public dental care (-2.7% points, -4.7%, *p* =.017) and increased the average amount of out-of-pocket spending on private care (12.1 euros, 29.8%, *p* =.032). The results suggest that, even in a country with a universal public dental care coverage, changes in cash transfers do affect the dental care patterns of low-income populations.

**Supplementary Information:**

The online version contains supplementary material available at 10.1186/s12913-024-10933-0.

## Introduction

Oral diseases create a significant health burden for many people around the world by lowering the quality of life and causing pain, discomfort, and even death [1, 2]. In addition, oral diseases share common risk factors, and they are associated with many other major diseases [3–8]. Most oral health conditions are largely preventable and can be treated in their early stages. However, for individuals, treatments for oral health conditions are often expensive even in countries with universal health care coverage. In high income countries, dental treatments comprise as much as 20% of out-of-pocket health expenditure, on average [9].

Oral diseases affect individuals within societies unequally. Earlier research has indicated a strong and consistent association between socioeconomic status, measured as income, occupation, and educational level, and the prevalence and severity of oral diseases [10–13]. Lifestyle factors and quality of nutrition obviously contiribute to dental health. Part of the socioeconomic inequality in dental health may, however, result from differences in the use of dental health care services. Previous research has shown that despite having poorer dental health, lower socioeconomic groups utilize less dental care services [14–18]. Sosioeconomic status predicts the use of dental care even in countries with publicly subsidized universal health care, such as Finland [19, 20].

This study examines the effect of a cash transfer reform on the use of dental care services. Although the socioeconomic gradient in the use of dental services is well documented, we lack experimental evidence on the effects of income on dental care usage. The study contributes to the topic by analyzing the use of dental care services in the Finnish basic income experiment. The experiment introduced an exogenous increase in the income of previous basic unemployment benefit recipients for a period of two years in 2017–2018. The probability of visiting, number of visits, and out-of-pocket costs of dental care services are analyzed with data collected from administrative registers. If dental care costs create a barrier to meet the needs of dental care among low-income groups, as documented in other contexts [21–24], increasing cash transfers should lead to an increase in the overall use of dental care services.

In a broad perspective, the study contributes to the research on the relationship between income and health by examining one specific causal pathway, i.e., the effect of an income change on the use of health services. From a policy perspective, the study aims to increase our understanding on the role of cash transfers in meeting the dental care needs of low-income populations in a universal and publicly subsidized health care system.

### Dental care usage in previous policy experiments

Previous studies have indicated that persons with lower income tend to use less dental care services than persons with higher income, even though the needs of the former may be greater than those of the latter [14–19, 25, 26]. However, only a few studies have examined the causal effect of changes in individuals’ economic conditions on dental care usage in a controlled setting.

Experimental studies in low-income and middle-income countries have found that the introduction of a cash transfer program that improves individuals’ economic resources may increase the use of preventive health care [27]. However, evidence on the comparable effectiveness of different types of cash transfer reforms remain weak [28]. Moreover, dental care usage spesifically has not been measured in the studies.

Most of the experimental research conducted in high-income countries on the effects of policies on health care usage focus on different types of insurance schemes. For example, in the RAND Health Insurance Experiment, reducing the patient’s part in cost-sharing increased the demand for dental services during the first year of the policy change [29]. In the Oregon Health Insurance Experiment, Medicaid insurance coverage significantly reduced the share of respondents reporting unmet dental care needs and doubled the share of people visiting the emergency departments for dental care, but it had no detectable effect on out-of-pocket spending [30]. In another study, extending dental coverage provided through a health insurance program for children in Western Pennsylvania increased their access to both dental care and preventive dental services [31].

Only a few studies conducted in high-income countries have assessed the dental care effects of direct cash transfers. In the Family Rewards Experiment, introducing a cash transfer program conditional to using health services led to a consistent and large increase in the use of preventive dental care [32]. On the effects of unconditional cash transfers, the evidence base is less clear [33]. In the Iowa and North Carolina Rural Income Maintenance Experiment (RIME), small but inconsistent effects on the use of health services, including dental care consumption, were found [34]. Relatively recently, a study that interviewed program participants in the Ontario Basic Income Pilot (OBIP) indicated that some low-income participants used their additional income on health services, including dental treatments [35].

To sum up, experimental studies on the dental care effects of cash transfer programs that are not conditional to using health services are rare. Available studies are based on uncontrolled study designs or report inconsistent findings. In addition, studies on cash transfer reforms in the context of universal health care systems are basically non-existent. Studying dental care usage in the Finnish basic income experiment provides a unique opportunity to contribute to the knowledge gap– with register-based data from a randomized field experiment that introduced an exogenous increase in the income of previous basic unemployment benefit recipients for a period of two years.

### Institutional context and the policy experiment

#### The Finnish dental care system

In Finland, dental care is organized through both public and private health care schemes. Dental care provided by the public sector is universally covered for all residents. Until 2023, municipalities provided basic dental care including dental checks and follow-up checks. Specialized dental care was provided by both the municipalities and the hospital districts. Public services are mainly financed through taxation. However, co-payments are usually required for dental appointments and procedures. In 2018, the maximum basic co-payment for an adult for a visit to a public dentist was 13.10 euros [36]. The out-of-pocket costs for different dental procedures varied between 8.40 euros and 183.50 euros. The annual ceiling for co-payments of public health care services was 683 euros. In addition, low-income households may receive basic social assistance to cover the costs of public oral and dental care services.

Dental care provided by the private sector is based on a free-market principle [37]. The average price for a basic oral examination in the private sector was 63 euros in 2018 [38]. The use of private dental care is partly subsidized through the National Health Insurance scheme. It covers dental checks, treatments, and laboratory and X-ray examinations but excludes cosmetic procedures and prosthodontics. In addition, procedures conducted by a dental hygienist may be covered. In 2018, the total reimbursement rate for dental care was 14.2% [39]. The National Health Insurance coverage and reimbursement rate have decreased over the years. Since 2016, dental examinations have been reimbursed under the National Health Insurance scheme only every other year, unless the patient’s health status, as verified by a dentist, requires more frequent examinations.

In Finland, dental care provided by the public sector has been associated with long waiting times. In October 2018, 45% of the patients with non-urgent appointments in public dental care had waited longer than three weeks [40]. Waiting times are typically shorter in the private sector, and the patient can choose the dentist freely. It is typical for private dentists to offer their patients annual or biannual recalls. Due to lack of resources, public dentists mostly use regular recalls for children [41].

Limited availability of public care, especially in non-urgent cases, may channel service demand to the private market.

In addition, previous research has shown that socioeconomic background predicts the dental care provider in Finland. Those with the lowest income use public services more frequently than private services. The higher the income and the higher the education, the more likely a person is to use private services [20]. To account for the specificities of the Finnish dental care system, this study collects register data on the use of both public and private dental care services and examines both the overall use and use by service provider.

#### The basic income experiment

During 2017–2018, the Government of Finland conducted a field experiment that tested an unconditional cash transfer policy in practice. The experiment was targeted at 25–58 year-old persons who received basic unemployment benefits from the Social Insurance Institution in November 2016 (*N* = 175,222). The intervention in the experiment was a specific change in the social benefit legislation: The persons chosen to participate in the experiment received an unconditional cash transfer (a basic income) of 560 euros per month without an obligation to search for a job, make benefit claims, or report earnings. In addition to replacing a part of the existing social benefit schemes with an unconditional cash transfer, the intervention increased monetary incentives of finding a job during the experiment because basic income payments were paid also for the employed. The experiment was designed as a randomized field experiment. From the target population, 2,000 persons were randomly chosen to be in the basic income group for the duration of two years. The rest of the target population formed the control group.

The primary objective of the experiment was to evaluate the effect of an unconditional cash transfer policy on the recipients’ labor market behavior. According to the main evaluation study, the experiment had no effect on employment during the first year. During the second year, persons in the basic income group had slightly more days in employment (6.6) than persons in the control group [42]. As a result of the experiment, however, the average annual income increased significantly in the basic income group. In the average annual income, including taxable market income, taxable social benefits, basic income, and housing benefits, there was an increase of 9.2% (1,330.1 euros) during the first year and 11.0% (1,734.7 euros) during the second year [42]. The increase in income was mostly due to structural changes attributable to the intervention: First, unemployment benefits from December 2016 and the first basic income payments were received in January 2017, doubling the benefit income for most of the participants in the beginning of the experiment. Second, persons who found a job before or during the experiment received basic income payments in addition to their earnings. Part of the increase during the second year (15.6%) was also due to behavioral changes in the labor market, i.e., the slight employment effect contributing to higher earnings in the basic income group compared to the control group during the second year [42].

## Methods

### Study design

To enable causal inference about the average treatment effect of the Finnish basic income experiment on the use of dental care services, the original experimental design of the experiment is exploited by comparing the study outcomes of the marginally randomized basic income group and control group. Because persons were randomly allocated to the basic income group and to the control group, the groups are similar both in their observed and unobserved characteristics. Thus, any differences in the use of dental care services are attributable to the policy intervention. Being in the basic income system versus being in the existing tax-benefit system is the only characteristic that differs systematically between the study groups.

### Data sources

For the target population of the Finnish basic income experiment, the data were collected from administrative registers for the years 2015–2019 and linked together using pseudonymized individual identifiers. Demographic data, including information on gender, age, having children, having a partner, native language, and place of residence, were derived from the Benefit Register of the Social Insurance Institution. Information about the treatment status in the experiment and previous unemployment benefit type were collected from the Social Insurance Institution’s Basic Income Experiment Register. Annual-level information on taxable income was gathered from the Personal Income Tax Register. Register data on the utilization of public dental care services were obtained from the Register of Primary Health Care Visits (municipal health care centers) and from the Care Register for Health Care (units of hospital districts) maintained by the Finnish Institute of Health and Welfare. The registers contain information on the dates of visits and received treatments. Information on the use of private dental care services was collected from the Benefit Register of the Social Insurance Institution. This information covers the dates and costs of health care visits and treatments reimbursed by the National Health Insurance scheme. Reimbursed procedures include dental checks, treatments, and examinations but exclude cosmetic procedures and prosthodontics. Data on the use of public and private care was restricted to visits containing oral-health-related procedures defined in the national classification of oral health care procedures [43].

### Outcome variables

In the main analysis, the individual-level outcome variables were defined as (1) having one or more visits to dental care services, (2) the total number of visits to dental care services, and (3) the out-of-pocket expenditure on dental care services during the experimentation period (2017–2018). The large range of outcome variables was chosen in order to capture and differentiate between changes in access (yes/no), volume (number of visits), and consumption patterns (out-of-pocket spending). In addition, the study outcomes were separately defined for public (including primary care and hospital care) and private dental care services to take into account the institutional specificities of the Finnish dental care system.

A visit to dental care may contain several procedures. For this study, a visit was further defined as a single date with oral-health-related procedures, i.e., each person was set to have a maximum of one visit per day and per service provider (primary care, hospital care, private care). Concerning primary care services, the data were limited to contacts that were actual visits by using information on the contact type and excluded, for example, remote contacts by phone or email.

Data on dental care expenses were available only for the private service use. Out-of-pocket expenditure was defined as costs of dental procedures after National Health Insurance reimbursements. The expenses were operationalized as out-of-pocket costs in order to directly measure how much money the participants spend on private care services. In the main analysis, total private dental care expenditure is also reported to evaluate the impact on the National Health Insurance system.

For the outcome variables of the main analysis, a two-year period (2017–2018) was chosen for two reasons. First, it is typical for dentists to recommend booking a visit for regular checkups for every other year only. Second, in 2016, the National Health Insurance scheme was changed to cover visits in the private sector only every other calendar year unless the patient’s health status, verified by a dentist, requires otherwise.

For descriptive purposes, all outcome variables were calculated also for the two-year period preceding the experiment (2015–2016). For further analyses (see Supplement), annual and monthly outcome variables were calculated for the whole data period of 2015–2019. In addition, outcomes during the experiment (2017–2018) were calculated separately for selected dental procedure categories and for different service providers (private care, primary care, hospital care) to gain information on different types of services, such as preventive, specialized and emergency care.

Visits to private dental care services are not recorded in the data if a person does not apply for National Health Insurance reimbursements. However, the number of unreimbursed cases is likely very small because reimbursements are usually handled automatically when paying for the service. In addition, the use of dental care services provided by the employers is not measured in the data. On the other hand, dental care is rarely included in the health care service contracts of the employers, and thus the employer-provided visits are expected to be very few in the study population.

### Statistical approach

The study utilizes the following statististical approach: First, basic descriptives of the background variables of the basic income group and control group are reported in order to describe the study population and to evaluate the success of the randomization procedure in balancing the study groups in relation to characteristics that may predict the later use of dental care services. Second, the average treatment effects on different outcome variables are analysed by estimating the following Ordinary Least Squares (OLS) regression model:

$$ {Y}_{i}=\alpha +{X}_{i}^{{\prime }}\beta +\delta {Tr}_{i}+{\epsilon }_{i}$$

In the equation, $$ {Y}_{i}$$ represents the examined dental care usage outcomes (one or more visits, number of visits, out-of-pocket costs) measured for the two-year experimentation period 2017–2018. $$ {Tr}_{i}$$ is the treatment status indicator (basic income groups vs. control group), $$ {X}_{i}$$ includes the control variables for different background characteristics observed before the experiment, and $$ {\epsilon }_{i}$$ is the individual-level error term in the model.

The average treatment effect is estimated both with a simple (1) and a multiple (2) OLS regression model with heteroskedasticity-robust standard errors. In a marginally randomized experiment, a simple regression model (1) suffices for estimating the average treatment effect. However, adding baseline variables with predictive power as covariates in the model may increase the statistical power of the estimation [44]. Covariates in the multiple model (2) include previous unemployment benefit type, gender, age group, having children, having a partner, native language, urbanization level of the place of residence, and previous use of public and private dental care services.

In the multiple model (2), age is categorized into three brackets: 25-34, 35-44, and 45-59. Gender is included as a binary covariate, and native language is coded as official domestic language (Finnish or Swedish) or foreign language. Family structure is measured with the number of dependent children, recoded into a binary variable having or not having children, and with marital status and with information on cohabitation, together recoded into a binary variable having a partner (= married or cohabiting) or not having a partner. Data on the place of residence (municipality) is categorized to urban, semi-urban, and rural municipalities according to Statistics Finland’s classification for year 2016 [45]. For age, children, partner, and place of residence, information from the end of 2016 is used.

The estimation is complemented with an analysis of effect heterogeneity withing selected subgroups. The analysis is conducted by estimating the simple model (1) separately for each of the subgroups. In addition, regression analyses are conducted separately for visits with surgical and non-surgical dental care procedures and for visits with different types of non-surgical procedures (e.g., examinations, preventive procedures, and restorative treatments) (see Supplement).

### Study population descriptives

The target population of the Finnish basic income experiment composed of low-income individuals. Accoding to the main evaluation study, persons in the study population had 24 days in employment and 286 days in unemployment during year 2016, on average [42]. Average earnings from employment were 1,900 euros in 2016.

Table 1 describes the socioeconomic and demographic background characteristics of the study groups. The mean annual taxable income, including earnings and taxable social benefits, was around 10,800 euros in the basic income group and in the control group in 2016. The gender ratio was quite equal between the study groups, 48% of the persons being women. The mean age in the basic income group was 40.8 years and 40.4 years in the control group, and 25% of the persons in the groups had other than Finnish or Swedish (official domestic languages) registered as their native language. There are no statistically significant differences between the study groups regarding the listed background characteristics at 5% significance level indicating a successful randomization procedure in balancing the study groups.

**Table 1:** Background characteristics before experiment (2016)

	Basic income group	Control group	Difference	P-value
Annual taxable income, €	10744.43	10826.21	-81.78	0.413
Women, %	47.8	47.5	0.3	0.812
Age				0.294
25–34, %	33.5	35.1	-1.6	
35–44, %	27.4	27.1	0.3	
45–59, %	39.0	37.7	1.3	
Foreign language, %	24.6	25.4	-0.8	0.398
Place of residence				0.731
Urban, %	79.0	79.5	-0.5	
Semi-urban, %	10.9	11.0	-0.1	
Rural, %	10.0	9.5	0.5	
N	2000	173,222		

*Notes* For continuous and binary variables, *p*-values are based on t-tests. For categorical variables, *p*-values are based on chi2-tests

In order to further describe the study population, Table 2 summarizes the pre-experimental use of dental care services in the study groups (a two-year period 2015–2016). In both groups, 63% of the persons had a visit in dental care services. About 56% visited primary care, while 6% in the basic income group and 5% in the control group had visits in hospital care. Approximately 11% in both groups used private dental care services.

**Table 2:** Use of dental care before the experiment (2015–2016) by study group

	Basic income group	Control group	Difference	P-value
Visiting				
Any care, %	62.5	62.5	-0.1	0.957
Primary care, %	55.5	55.9	-0.4	0.730
Hospital care, %	6.1	5.3	0.8	0.121
Private care, %	11.1	10.9	0.2	0.781
Number of visits to				
Any care	5.86	5.84	-0.02	0.927
Primary care	5.03	5.06	-0.03	0.857
Hospital care	0.14	0.16	-0.02	0.368
Private care	0.70	0.63	0.07	0.302
Out-of-pocket expenditure				
Private care, €	44.49	40.87	3.62	0.445
N	2000	173,222		

*Notes P*-values are based on t-tests

Number of visits to dental care services during the two-year period was 5.9 in the basic income group and 5.8 in the control group, on average. In the basic income and control groups, the number of visits in primary care was 5.0 and 5.1, in hospital care 0.1 and 0.2, and in private care 0.7 and 0.6, respectively. Persons in the basic income group spent, on average, 44 euros in private dental care services during the two years before the experiment, while the average expenditure in the control group was 41 euros. The differences between the study groups are not statistically significant at 5% significance level indicating balanced study groups needed for a design-based causal inference.

### Results: effects on the use of dental care services

Table 3 reports the estimated average treatment effects on the selected outcome variables measuring the use of dental care, i.e., the probability of visiting, total number of visits, and the out-of-pocket expenditure on dental care services. The estimation period covers the whole two years of the experiment (2017–2018), and the effect estimates are provided separately for using any care, public care (including primary care and hospital care), or private care. Table 3 reports the estimates from a simple OLS regression with only the treatment status indicator as a predictor.

**Table 3:** Average effects on the use of dental care (2017–2018)

	Control group mean	Estimate	S.E.	P-value
Any care				
P of visiting	0.634	-0.016	0.011	0.132
Visits	5.34	-0.08	0.17	0.627
Public care				
P of visiting	0.570	-0.027	0.011	0.017
Visits	4.76	-0.19	0.16	0.212
Private care				
P of visiting	0.114	0.013	0.007	0.072
Visits	0.57	0.12	0.06	0.066
Total expenditure (€)	47.56	13.83	6.43	0.032
Out-of-pocket expenditure (€)	40.58	12.09	5.65	0.032

*Note* Using simple linear Ordinary Least Squares regression (i.e., only treatment status indicator as a predictor) and heteroskedasticity-robust standard errors for all outcomes

Based on the estimation, we do not find statistically significant effects (at 5% level) on the overall use of dental care services, measured both in probability of visiting and total number of visits. However, we do find a statistically significant negative effect of -2.7% points (-4.7% in relative terms) on the probability of visiting public care (*p* =.017). The estimated effect on the number of visits to public care is also negative, -0.2 (-4.2%), although the estimate is not statistically significant (*p* =.212).

The estimated effect on the probability of visiting private care is positive, 1.3% points (11.9%), as is the estimated effect on the number of visits to private care, 0.1 (20.4%), but neither of the estimates is statistically significant (*p* =.072 and *p* =.066, respectively). However, we find a statistically significant positive effect of 12.1 euros (29.8%) on the out-of-pocket expenditure on private care (p.=0.032). The effect estimate on total expenditure (before National Health Insurance reimbursements) indicates a proportional effect on the National Health Insurance expenditure. Adjusting for the selected background characteristic in the regression produces effect estimates and standard errors of similar sizes as found in Table 3 (see Table S1 in Supplement).

In sum, we do not find a statistically significant effect on the use of dental care services overall. However, the estimation indicates a negative effect on the use of public dental care and a positive effect on the use of private care. Further graphical examinations and statistical estimations provided in Supplement support the findings of the main analysis.

## Discussion

The results of the study indicate that the patterns of using dental care services among low-income populations may be affected by changes in the policies that directly impact individuals’ economic circumstances, even in a country with a universal public dental care coverage. The found positive effect on the use of private care fits the hypothesis that increasing economic resources should lower the cost barrier for using dental care services among low-income populations [21–24]. However, the lack of evidence about a positive effect on the overall use and the found negative effect on the use of public care suggest that, instead of overall use, an increase in income may be affecting the individuals’ choice over the service provider. For example, given the potentially long waiting times in the public care, choosing private care may be an attractive choice when there are additional economic resources available [46].

The reported effect estimates on private dental care usage would be biased if the persons in the basic income group, having higher income than the control group, were less likely to apply for National Health Insurance reimbursements than the persons in the control group. In such case, the true effect of being in the basic income group on the use of private dental care would be underestimated. In addition, if the persons in the basic income group were more likely to use dental care services provided by the employers than the persons in the control group, then the effects on the overall use of dental care would be underestimated. The latter source of bias could partly result from the slight positive employment effect found during the second year of the experiment [42]. Increased use of employer-provided dental care services in the basic income group could also partly explain the reduction in the use of public services. However, asymmetric changes in the claiming of reimbursements and in the use of employer-provided dental services are unlikely, as, in Finland, National Health Insurance reimbursements are basically automatically provided when paying for private services, and employers only rarely have dental care services included in their occupational health care contracts.

In principle, instead of being direct effects of income the changes in the use of dental care services could partly be explained by changes in the participants’ dental health. Increasing income could lead to nutritional choices that are beneficial for the overall health, but the effect could also be detrimental [35, 47–49]. A survey study conducted near the end of the Finnish basic income experiment suggests that the experiment may have had positive effects on the participants’ self-evaluated state of health [50]. However, the short timespan (one year) between the policy change and the observed effects on the service-use makes it plausible that the changes in care-seeking behavior are a direct result of higher income rather than a result mediated by a genuine change in dental health.

## Conclusions

The study assessed the effect of cash transfers on the use of dental care services among a low-income population by utilizing register-based data from the Finnish basic income experiment (2017–2018). The experiment exogenously increased the annual income of the participants by 9.2–11.0% [42]. The study found no effects on the overall use of dental care services during the two-year experiment, but the results indicate that the experiment decreased the use of public care and increased the use of private care slightly. The probability of visiting public care decreased by 2.7% points (-4.7%, *p* =.017), the probability of visiting private care increased by 1.3% points (11.9%, *p* =.072), and the out-of-pocket expenditure on private care increased by 12.1 euros (29.8%, *p* =.032), on average. When comparing the change in expenditure to the increase in income caused by the intervention, private dental care consumption was more than proportionally affected.

The results of the study indicate that in a country with publicly provided universal dental care, increasing cash transfers does not necessarily increase the overall use of dental services among low-income populations. However, additional economic resources may affect an individual’s choice over the service provider. From the policy perspective, the findings encourage putting attention to the availability of services in meeting the needs of dental care among low-income populations: For example, lowering co-payments of public services may not be the most effective policy action to increase access if the availability of public care remains limited [20]. On the other hand, if the public services are under-resourced, then improving the economic resources of a low-income population or reducing the patient’s part in cost-sharing of private services could serve as an immediate policy action to direct service demand from the public sector to the private providers. Obviously, the findings are likely highly context-specific, and the overall conclusions about the relative importance of individuals’ economic resources, cost of services, and the service supply in meeting the dental care needs of low-income populations may differ in other countries [51].

Regarding the potential unmet dental care needs of low-income populations, more money does not necessarily lead to more use overall, but it may give more freedom in choosing the service provider. If changing service providers means getting the needed dental care earlier, then at least some pain and discomfort may be avoided leading to higher quality of life and, in the best case, improved overall health.

### Electronic supplementary material

Below is the link to the electronic supplementary material.

Supplementary Material 1

## Data Availability

Access to data that support the findings of this study was authorized by permissions from the officials that administer the registers. As a general rule, legal restrictions prevent the public sharing of sensitive pseudonymized data [54, 55]. In addition, the researchers’ permissions from the data providers do not allow data sharing. In principle, the data are available from the Social Insurance Institution of Finland, Finnish Tax Administration, and Finnish Institute of Health and Welfare, but restrictions apply to the availability of these data, which were used under license for the current study.
