# Supplementary material for: How an increase in income affects the use of dental care services among a low-income population: evidence from the Finnish basic income experiment
Source: BMC Health Serv Res. 2024 Apr 22;24:499. doi: 10.1186/s12913-024-10933-0 (PMC11036558; doi:10.1186/s12913-024-10933-0)
Supplement: Supplementary file 1 — Supplementary Material 1 [file 12913_2024_10933_MOESM1_ESM.docx]

A supplement to “How an increase in income affects the use of dental care services among a low-income population:

Evidence from the Finnish basic income experiment”

Contents

[Additional analyses 2](#_Toc155261407)

[Supplementary figures and tables 5](#_Toc155261408)

## Additional analyses

The found increase in the expenditure on private dental care is consistent with the hypothesis that increasing income may lower the cost barrier in the use of dental care services among a low-income population. Regarding the cost barrier assumption, however, no effects on the use of public dental care were found. Moreover, the effect estimates on the overall use of dental care and on the use of public dental care are actually negative, although only the latter is statistically significant. To further explore the use of dental care services in the Finnish basic income experiment, additional analyses were conducted.

Monthly shares of persons using dental care services indicate that the use of primary care remains at the same or lower level in the basic income group compared to the control group throughout the experimentation period 2017–2018 (Figure S1 in the Supplement). On the other hand, the lower level in the hospital care and the higher level in the private care both emerge close to the end of the first year of the experiment (Figure S2, and S3). Annual shares of persons using dental care services provide a similar story (Table S2), i.e, lower level for hospital care (-0.9 percentage points, -21.7%, p=.024) and higher level for private care (1.2 percentage points, 16.4%, p=.051) in the basic income group compared to the control group during the second year. Annual comparisons also show that the reduction in the use of primary care is larger during the first year (-1.6 percentage points) than during the second year (‑0.9 percentage points), although the annual differences in the use of primary care are not statistically significant. The use of hospital care remains lower (‑0.8 percentage points, ‑19.1%, p=.045) in the basic income group compared to the control group also during the year after the experiment (2019).

Analysis of effect heterogeneity among selected subgroups shows consistent results with the main analysis, although most of the effect estimates are not statistically significant (Table S3, S4, and S5). Estimates on the use of public care are either statistically not significant or negative for all group divisions. In contrast, estimates on the use of private care are either statistically not significant or positive for all group divisions. In almost all group divisions, estimates on the overall use are statistically not significant.

The heterogeneity analysis reveals some variation in the magnitude of the effect among different subgroups regarding different service providers (Figure S7, and S8). For example, among men, the number of visits to public care decreases less and the number of visits to private care increases more than among women. However, the confidence intervals of the effect estimates overlap between basically all group divisions.

Further analysis of the effects of the experiment on the probability of having different types of dental procedures during the experimental period fits the pattern found in the main analysis (Table S6). The effect on the probability of having surgical oral procedures is not statistically significant for public or private care. The probability of having non-surgical oral procedures in the public care is -1.5 percentage points lower in the basic income group than in the control group, but the effect estimate is not statistically significant (p=.194). On the other hand, the probability of having non-surgical oral procedures in the private care increases by 1.7 percentage points, and the estimate is statistically significant (p=.021). The effect estimate on the probability of having examinations in the public care is -1.3 percentage points, but the estimate is not statistically significant (p=.241). However, we find a statistically significant positive effect on the probability of having examinations in the private care (1.5 percentage points, p=.013). The magnitude of the effects on restorative treatments are of similar sizes, -1.9 percentage points for public care and 1.1 percentage points for private care, but the estimates are not statistically significant (p=.059 and p=.066, respectively). Effect estimates on the probability of having preventive procedures, treatments of periodontal diseases, and root canal treatments are not statistically significant for either service provider.

To summarize, the additional analyses on the use of dental care services in the Finnish basic income experiment support the main findings: While the level of overall use of dental care remained unaffected, the level of public care usage decreased and the level of private care usage increased slightly because of the intervention.

## Supplementary figures and tables


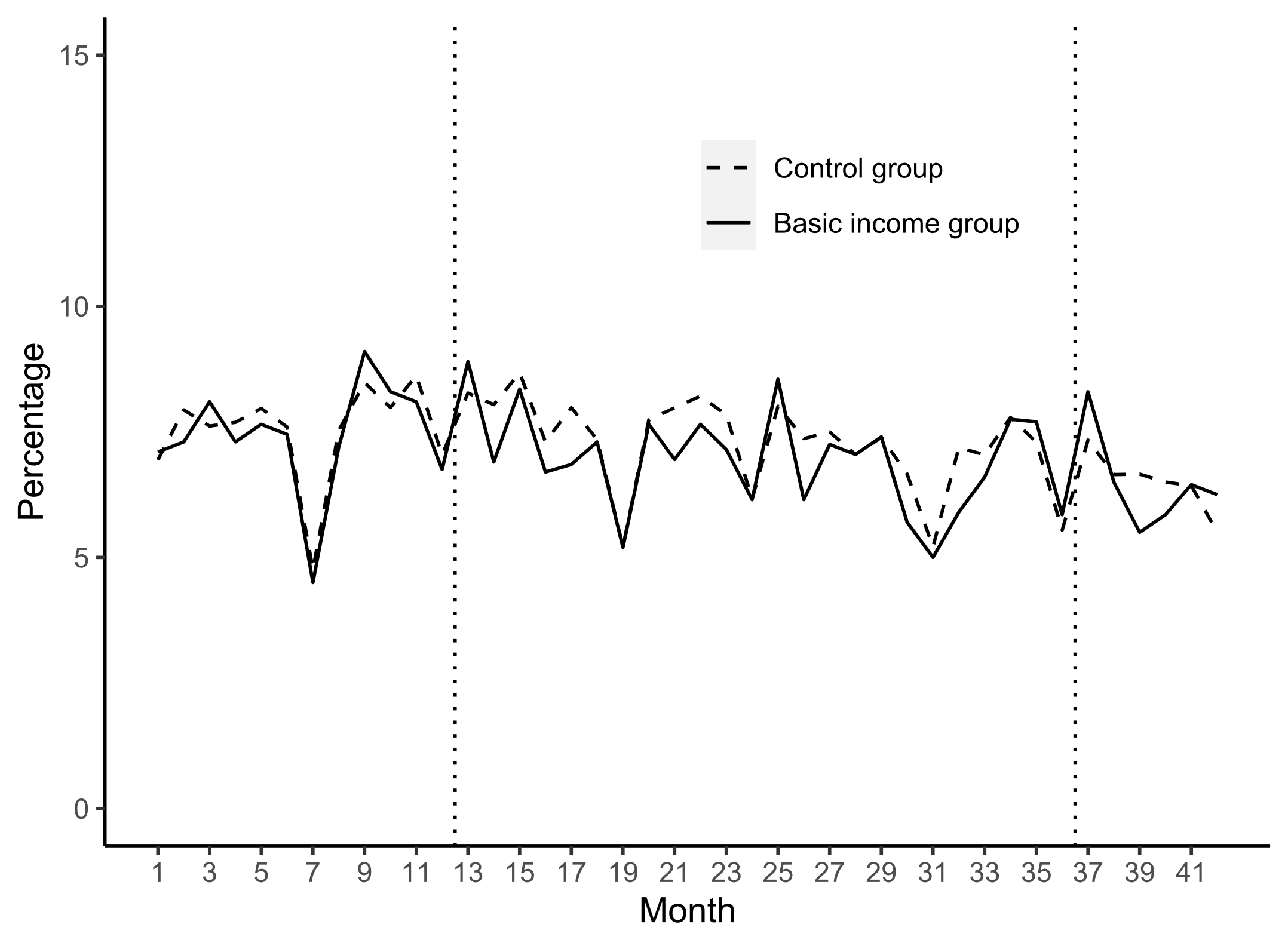


Figure S1. Share of persons visiting primary care, by month and by study group, during Jan 2016–Jun 2019.


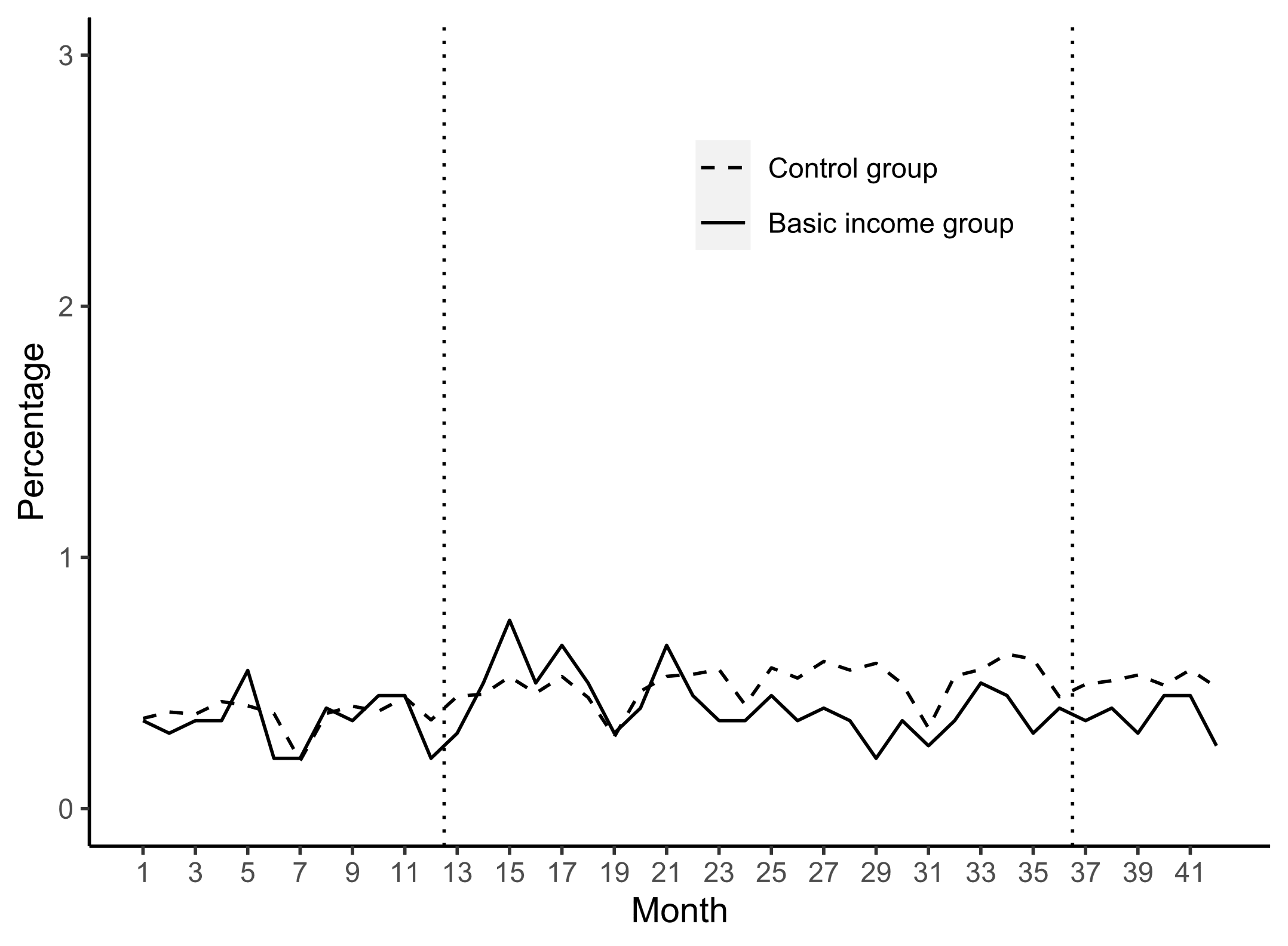


Figure S2. Share of persons visiting hospital care, by month and by study group, during Jan 2016–Jun 2019.


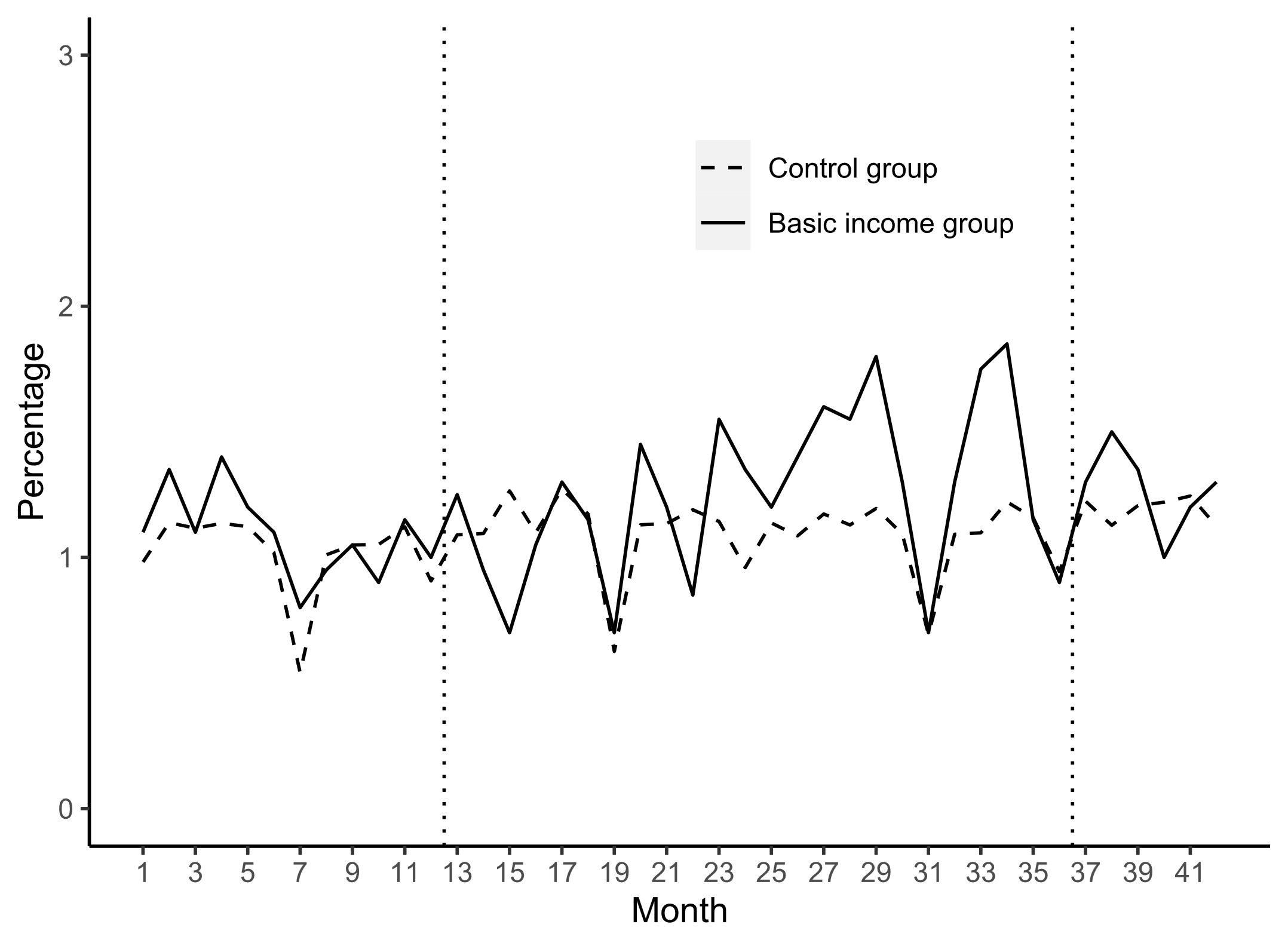


Figure S3. Share of persons visiting private care, by month and by study group, during Jan 2016–Jun 2019.


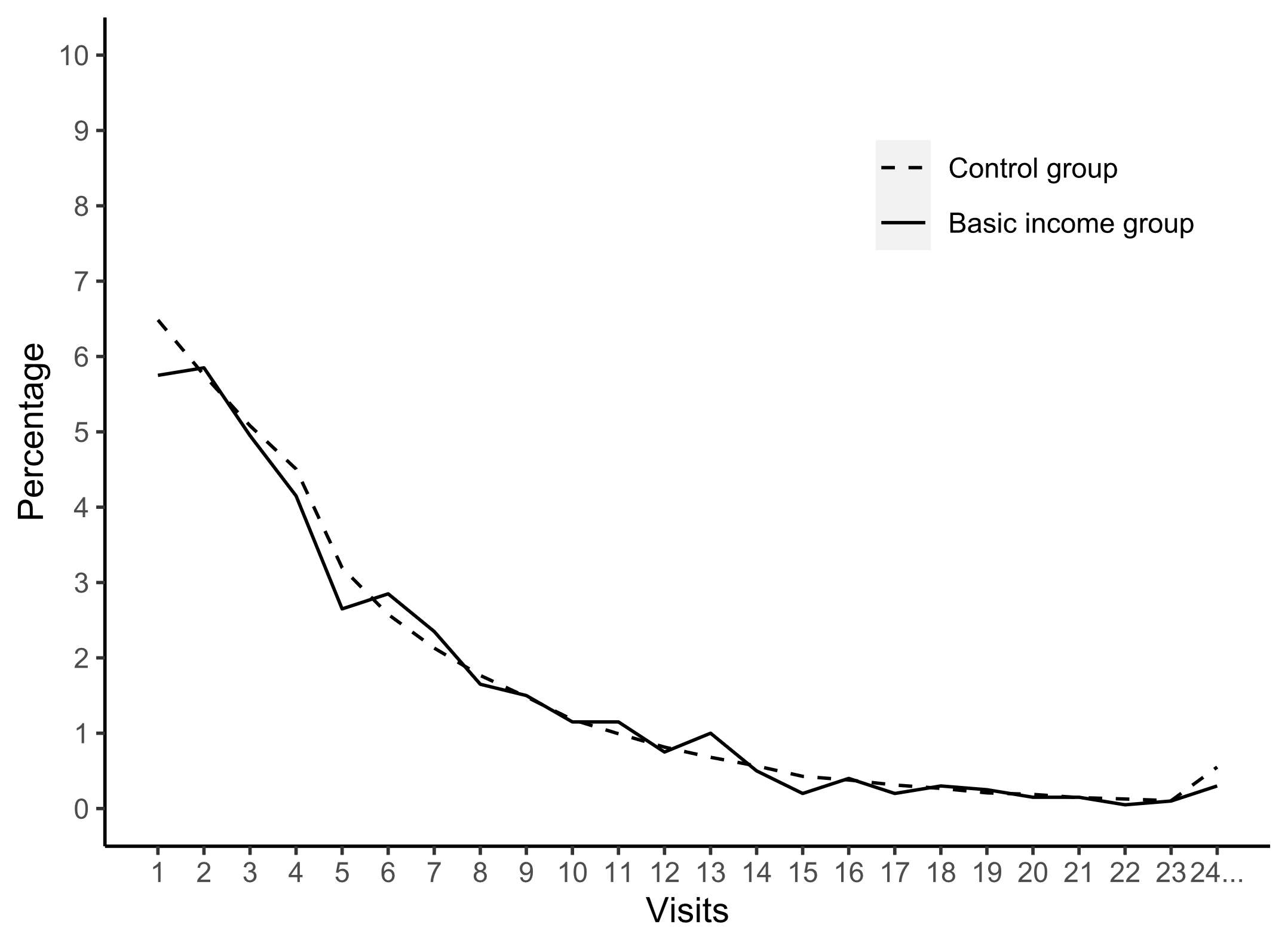


Figure S4. Distribution of visits to public care in the basic income group and control group, during experiment (2017–2018). Zero visits not shown in the graph.


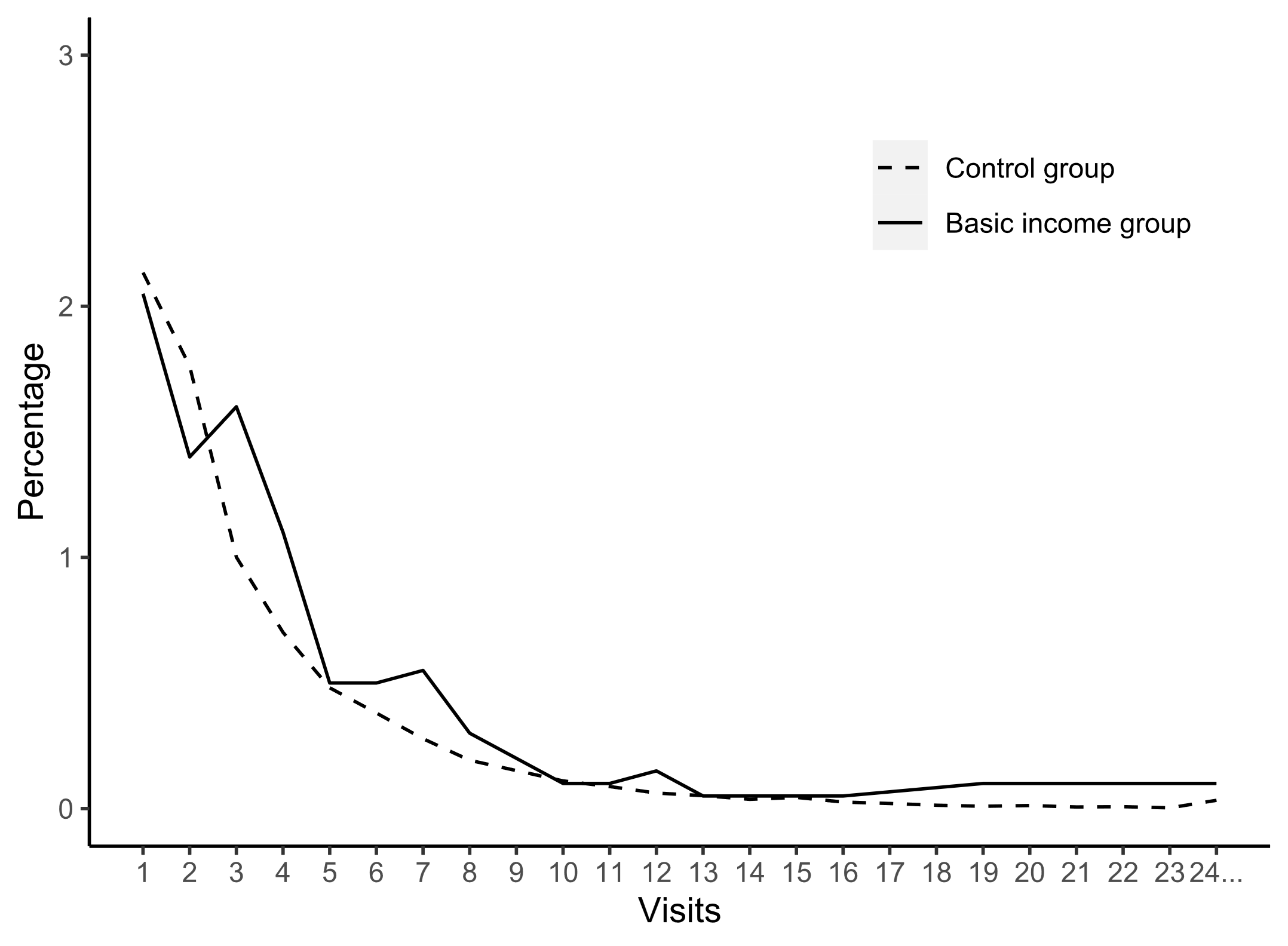


Figure S5. Distribution of visits to private care in the basic income group and control group, during experiment (2017–2018). Zero visits not shown in the graph.


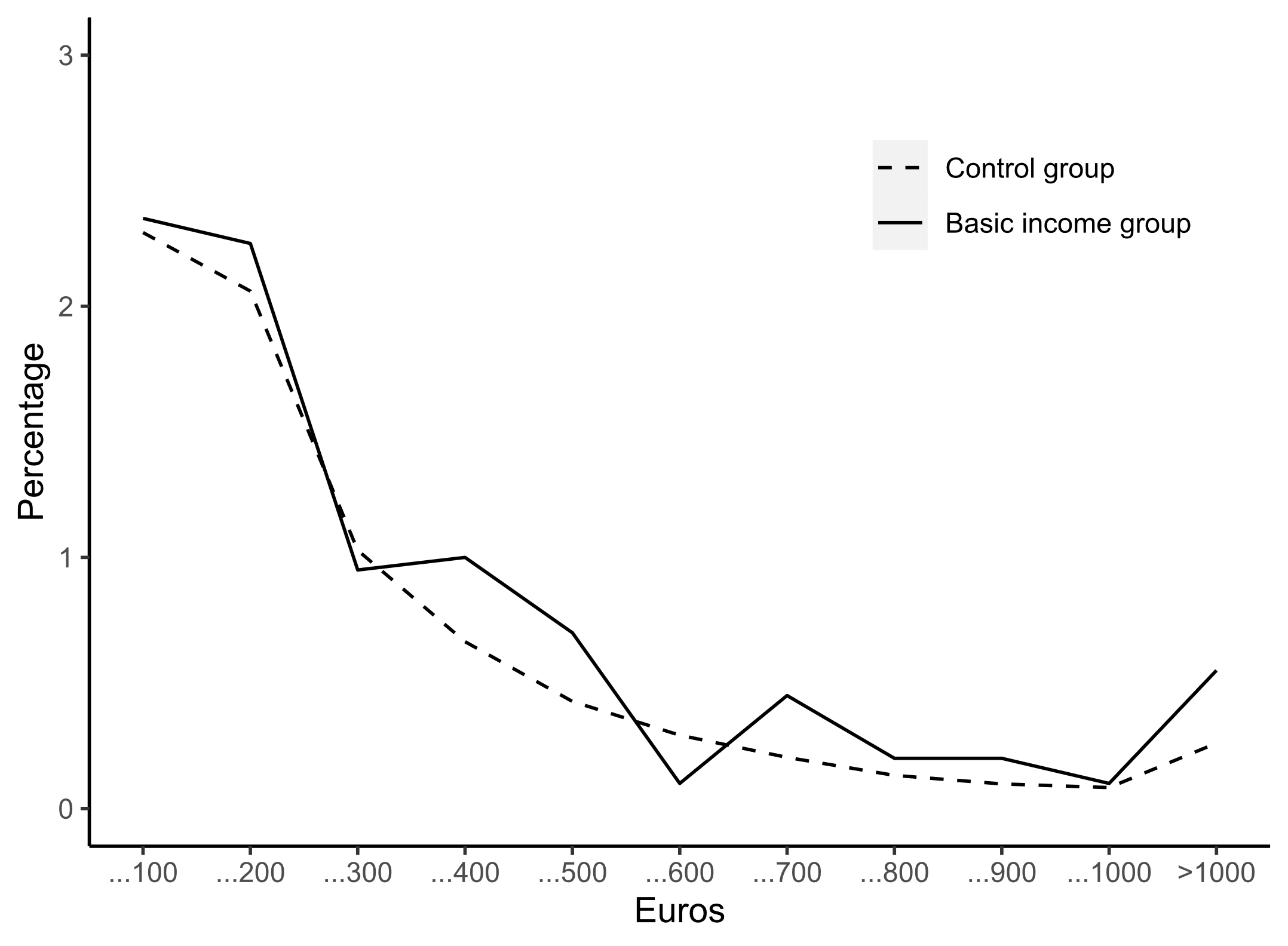


Figure S6. Distribution of out-of-pocket expenditure on private care in the basic income group and control group during experiment (2017–2018). Zero expenditure not shown in the graph.

Table S1. Average effects on the use of dental care estimated with simple and multiple linear regression models (2017–2018).

|  | Control mean | Model 1 |  |  | Model 2 |  |  |
| --- | --- | --- | --- | --- | --- | --- | --- |
|  |  | Estim. | S.E. | P-val. | Estim. | S.E. | P-val. |
| Any care |  |  |  |  |  |  |  |
| P of visit | .634 | -.016 | .011 | .132 | -.017 | .010 | .093 |
| Visits | 5.34 | -0.08 | 0.17 | .627 | -0.09 | 0.16 | .590 |
| Public care |  |  |  |  |  |  |  |
| P of visit | .570 | -.027 | .011 | .017 | -.026 | .010 | .012 |
| Visits | 4.76 | -0.20 | 0.16 | .212 | -0.19 | 0.15 | .205 |
| Private care |  |  |  |  |  |  |  |
| P of visit | .114 | .013 | .007 | .072 | .012 | .007 | .077 |
| Visits | 0.57 | 0.12 | 0.06 | .066 | 0.11 | 0.06 | .080 |
| Costs (€) | 40.58 | 12.09 | 5.65 | .032 | 11.24 | 5.42 | .038 |

Note: Using Ordinary Least Squares regression and heteroskedasticity-robust standard errors for all outcomes. Model 1=simple linear regression incl. only treatment status indicator as a predictor, Model 2=multiple linear regression incl. dummy variables for background characteristics (previous unemployment benefit type, gender, age group, having children, having a partner, native language, urbanization level of the place of residence, and previous use of public and private dental care services).

Table S2. Group differences in the probability of visiting dental care annually during 2015–2019 (experimentation period 2017–2018).

|  | Control mean | Difference | S.E. | P-val. |
| --- | --- | --- | --- | --- |
| 2015 |  |  |  |  |
| Primary care | .370 | .004 | .011 | .708 |
| Hospital care | .029 | .005 | .004 | .237 |
| Private care | .077 | .006 | .006 | .312 |
| 2016 |  |  |  |  |
| Primary care | .396 | -.009 | .011 | .398 |
| Hospital care | .030 | .003 | .004 | .451 |
| Private care | .070 | .001 | .006 | .872 |
| 2017 |  |  |  |  |
| Primary care | .399 | -.016 | .011 | .141 |
| Hospital care | .037 | .002 | .004 | .634 |
| Private care | .076 | .004 | .006 | .499 |
| 2018 |  |  |  |  |
| Primary care | .379 | -.009 | .011 | .432 |
| Hospital care | .042 | -.009 | .004 | .024 |
| Private care | .076 | .012 | .006 | .051 |
| 2019 |  |  |  |  |
| Primary care | .341 | .014 | .011 | .194 |
| Hospital care | .043 | -.008 | .004 | .045 |
| Private care | .073 | .007 | .006 | .229 |

Note: Using simple linear Ordinary Least Squares regression (i.e., only treatment status indicator as a predictor) and heteroskedasticity-robust standard errors.

Table S3. Average effects on the probability of visiting any dental care within baseline subgroups during 2017–2018.

|  | Control mean | Estim. | S.E. | P-val. | N treated |
| --- | --- | --- | --- | --- | --- |
| All | .634 | -.016 | .011 | .132 | 2000 |
| Gender |  |  |  |  |  |
| Man | .575 | -.009 | .015 | .580 | 1045 |
| Woman | .699 | -.026 | .015 | .091 | 955 |
| Age |  |  |  |  |  |
| 25-34 | .630 | -.036 | .019 | .059 | 670 |
| 35-44 | .632 | .011 | .021 | .605 | 549 |
| 45-59 | .639 | -.019 | .017 | .275 | 781 |
| Having children |  |  |  |  |  |
| No | .610 | -.007 | .014 | .613 | 1153 |
| Yes | .666 | -.029 | .017 | .086 | 847 |
| Having a partner |  |  |  |  |  |
| No | .620 | .002 | .015 | .874 | 1049 |
| Yes | .650 | -.038 | .016 | .017 | 951 |
| Native language |  |  |  |  |  |
| Domestic (FI/SE) | .637 | -.015 | .013 | .227 | 1509 |
| Foreign | .626 | -.021 | .022 | .349 | 491 |
| Place of residence |  |  |  |  |  |
| Urban | .636 | -.022 | .012 | .072 | 1574 |
| Semi-urban | .636 | .002 | .033 | .962 | 218 |
| Rural | .613 | .007 | .035 | .832 | 200 |
| Previous service use |  |  |  |  |  |
| No visits | .438 | -.028 | .018 | .121 | 751 |
| Only public care | .742 | -.009 | .014 | .510 | 1027 |
| Only private care | .763 | -.009 | .037 | .799 | 138 |
| Both public/private | .842 | -.009 | .041 | .832 | 84 |

Note: Using simple linear Ordinary Least Squares regression (i.e., only treatment status indicator as a predictor) and heteroskedasticity-robust standard errors.

Table S4. Average effects on the probability of visiting public dental care within baseline subgroups during 2017–2018.

|  | Control mean | Estim. | S.E. | P-val. | N treated |
| --- | --- | --- | --- | --- | --- |
| All | .570 | -.027 | .011 | .017 | 2000 |
| Gender |  |  |  |  |  |
| Man | .521 | -.026 | .016 | .097 | 1045 |
| Woman | .624 | -.028 | .016 | .075 | 955 |
| Age |  |  |  |  |  |
| 25-34 | .576 | -.044 | .019 | .025 | 670 |
| 35-44 | .577 | -.009 | .021 | .688 | 549 |
| 45-59 | .559 | -.025 | .018 | .171 | 781 |
| Having children |  |  |  |  |  |
| No | .532 | -.022 | .015 | .141 | 1153 |
| Yes | .621 | -.033 | .017 | .052 | 847 |
| Having a partner |  |  |  |  |  |
| No | .560 | -.017 | .015 | .269 | 1049 |
| Yes | .581 | -.038 | .016 | .019 | 951 |
| Native language |  |  |  |  |  |
| Domestic (FI/SE) | .566 | -.027 | .013 | .039 | 1509 |
| Foreign | .581 | -.027 | .023 | .234 | 491 |
| Place of residence |  |  |  |  |  |
| Urban | .570 | -.030 | .013 | .019 | 1574 |
| Semi-urban | .576 | -.030 | .034 | .374 | 218 |
| Rural | .562 | -.007 | .035 | .849 | 200 |
| Previous service use |  |  |  |  |  |
| No visits | .395 | -.039 | .018 | .025 | 751 |
| Only public care | .726 | -.015 | .014 | .285 | 1027 |
| Only private care | .284 | -.030 | .037 | .418 | 138 |
| Both public/private | .656 | -.013 | .053 | .799 | 84 |

Note: Using simple linear Ordinary Least Squares regression (i.e., only treatment status indicator as a predictor) and heteroskedasticity-robust standard errors.

Table S5. Average effects on the probability of visiting private dental care within baseline subgroups during 2017–2018.

|  | Control mean | Estim. | S.E. | P-val. | N treated |
| --- | --- | --- | --- | --- | --- |
| All | .114 | .013 | .007 | .072 | 2000 |
| Gender |  |  |  |  |  |
| Man | .089 | .022 | .010 | .022 | 1045 |
| Woman | .140 | .003 | .011 | .773 | 955 |
| Age |  |  |  |  |  |
| 25-34 | .099 | .007 | .012 | .555 | 670 |
| 35-44 | .103 | .026 | .014 | .070 | 549 |
| 45-59 | .135 | .009 | .013 | .484 | 781 |
| Having children |  |  |  |  |  |
| No | .129 | .027 | .011 | .012 | 1153 |
| Yes | .093 | -.005 | .010 | .591 | 847 |
| Having a partner |  |  |  |  |  |
| No | .105 | .028 | .011 | .008 | 1049 |
| Yes | .123 | -.003 | .011 | .774 | 951 |
| Native language |  |  |  |  |  |
| Domestic (FI/SE) | .123 | .017 | .009 | .054 | 1509 |
| Foreign | .085 | 0.00 | .013 | .987 | 491 |
| Place of residence |  |  |  |  |  |
| Urban | .117 | .010 | .008 | .245 | 1574 |
| Semi-urban | .109 | .020 | .023 | .387 | 218 |
| Rural | .093 | .037 | .024 | .119 | 200 |
| Previous service use |  |  |  |  |  |
| No visits | .068 | .010 | .010 | .330 | 751 |
| Only public care | .056 | .015 | .008 | .061 | 1027 |
| Only private care | .624 | .035 | .041 | .384 | 138 |
| Both public/private | .421 | -.040 | .053 | .449 | 84 |

Note: Using simple linear Ordinary Least Squares regression (i.e., only treatment status indicator as a predictor) and heteroskedasticity-robust standard errors.


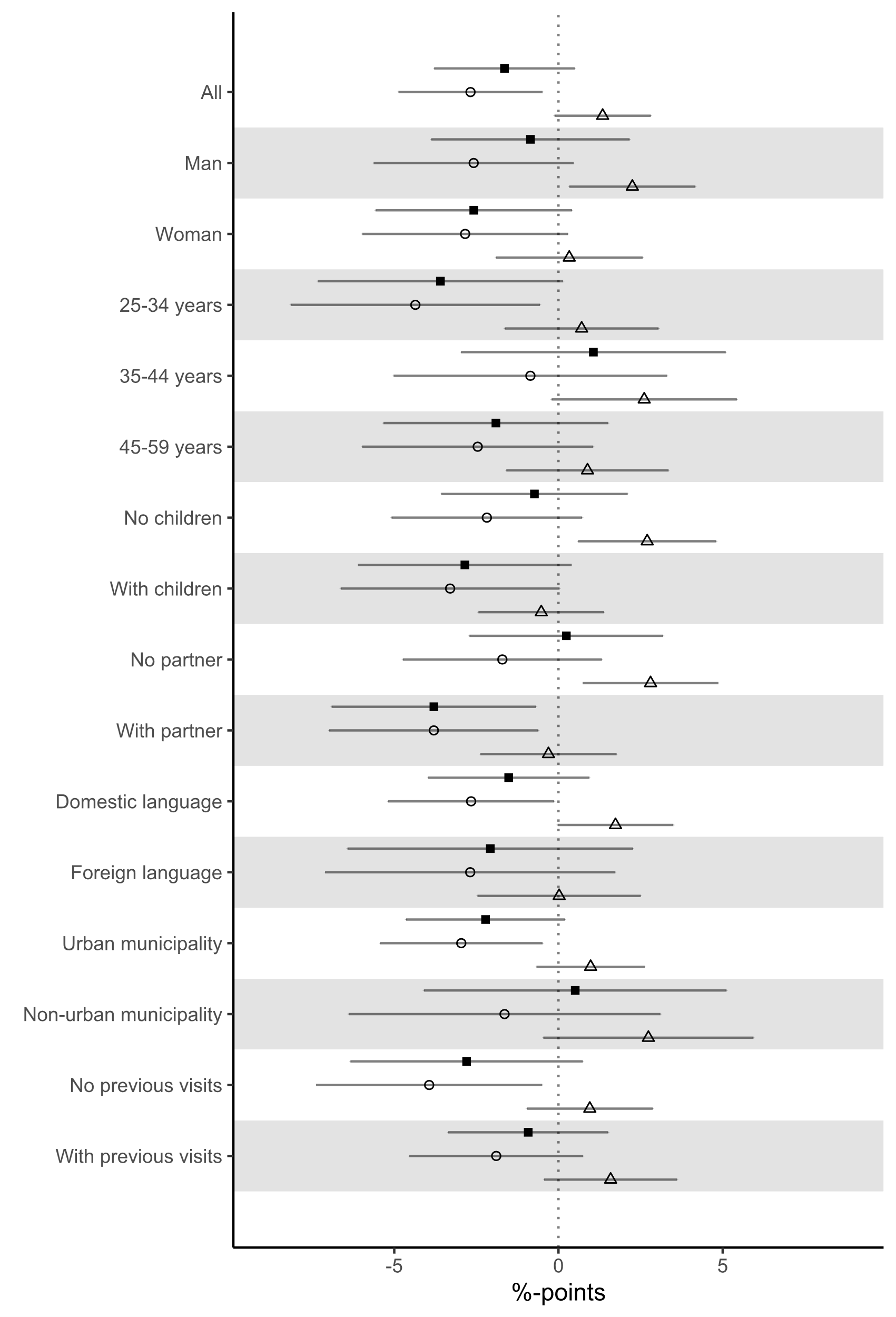


Figure S7. Average effects on the probability of visiting dental care within baseline subgroups and by service provider (square=any care, circle=public care, triangle=private care) during 2017–2018.


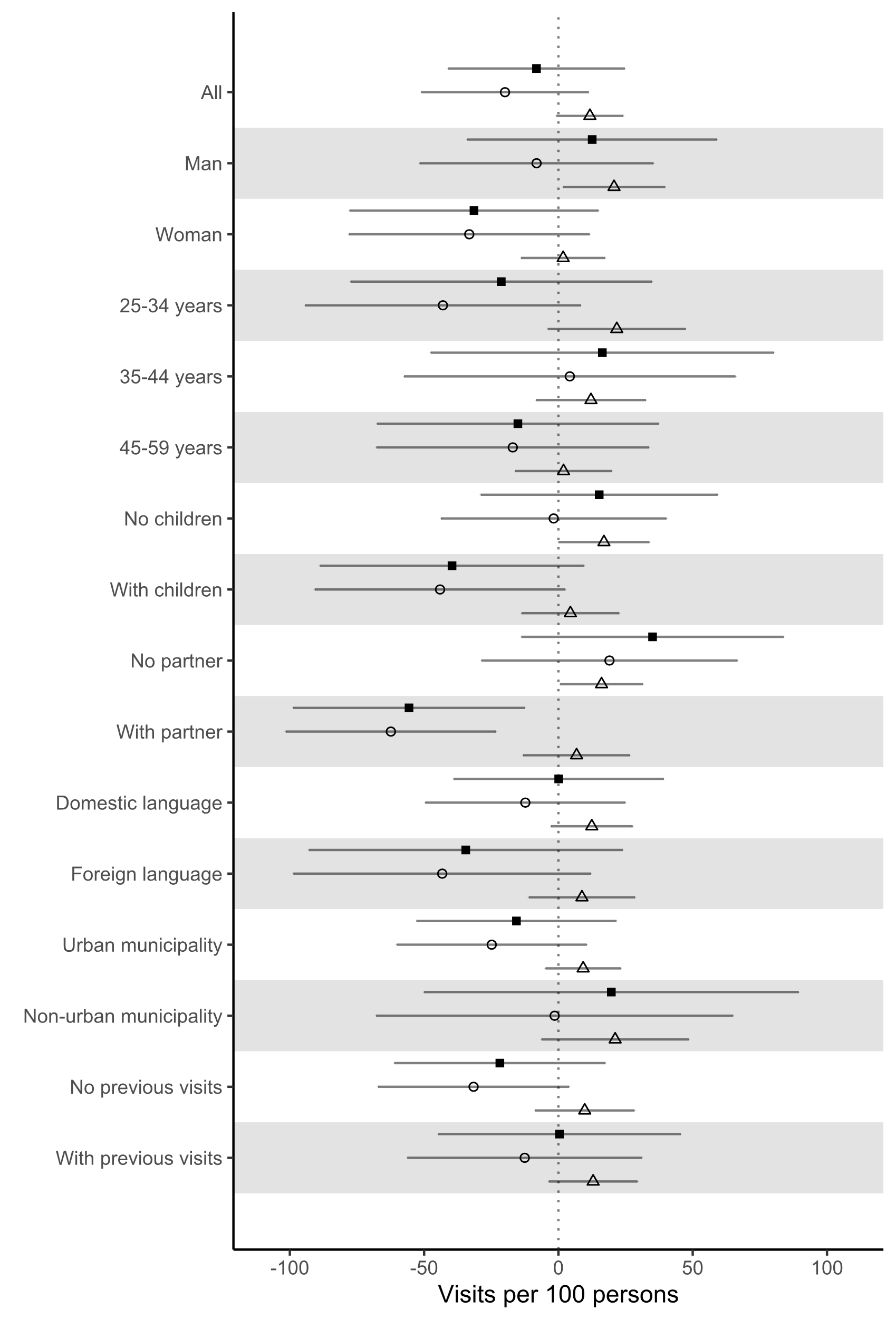


Figure S8. Average effects on the number of visits to dental care within baseline subgroups and by service provider (square=any care, circle=public care, triangle=private care) during 2017–2018.

Table S6. Group differences in the probability of visiting dental care during 2017–2018 by different types of dental care procedure.

|  | Control mean | Difference | S.E. | P-val. |
| --- | --- | --- | --- | --- |
| Surgical procedures of teeth, jaws, mouth and pharynx (E) |  |  |  |  |
| Public care | .364 | -.009 | .011 | .378 |
| Private care | .020 | .007 | .004 | .068 |
| Non-surgical procedures of mouth and teeth (S) |  |  |  |  |
| Public care | .492 | -.015 | .011 | .194 |
| Private care | .097 | .017 | .007 | .021 |
| Examination of mouth and teeth (SA) |  |  |  |  |
| Public care | .434 | -.013 | .011 | .241 |
| Private care | .061 | .015 | .006 | .013 |
| Oral health promotion, incl. preventive procedures (SC) |  |  |  |  |
| Public care | .057 | -.007 | .005 | .170 |
| Private care | .008 | .001 | .002 | .691 |
| Treatment of periodontal diseases (SD) |  |  |  |  |
| Public care | .235 | .000 | .010 | .968 |
| Private care | .055 | .004 | .005 | .403 |
| Restorative treatments (SF) |  |  |  |  |
| Public care | .309 | -.019 | .010 | .059 |
| Private care | .066 | .011 | .006 | .066 |
| Root canal treatments (SG) |  |  |  |  |
| Public care | .100 | -.002 | .007 | .776 |
| Private care | .014 | .001 | .003 | .730 |

Note: Using simple linear Ordinary Least Squares regression (i.e., only treatment status indicator as a predictor) and heteroskedasticity-robust standard errors.
